# Supplementary material for: Visualization of Bluetongue Virus RNA Segment Networks in Infected Cells: Multipartite Genomic RNA Assortment Is Independent of Viral Proteins NS2 and VP6
Source: Viruses. 2026 Mar 25;18(4):406. doi: 10.3390/v18040406 (PMC13120027; doi:10.3390/v18040406)
Supplement: Supplementary file 1 [file viruses-18-00406-s001.zip › viruses-4215110-supplementary.pdf]

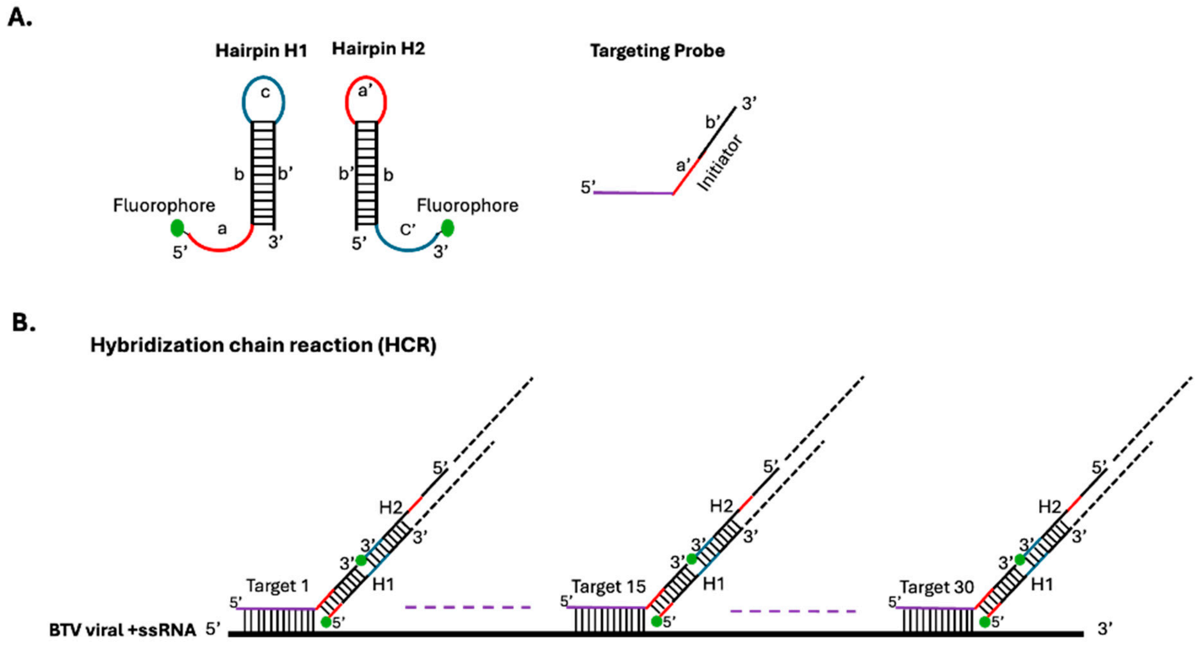

Figure S1. The cartoon shows the *in-situ* Hybridization Chain Reaction (HCR); previously published [13]. **(A)**The structures of H1 and H2 of hairpin DNA pairs, and corresponding targeting probe including initiator sequence. **(B)** The *in-situ* Hybridization Chain Reaction (HCR) strategy: the 30 targeting probes spanning the BTV viral +ssRNA segment, each including an initiator sequence hybridize to DNA hairpin H1 and then H1 and H2 hairpins continue to hybridize with each other.

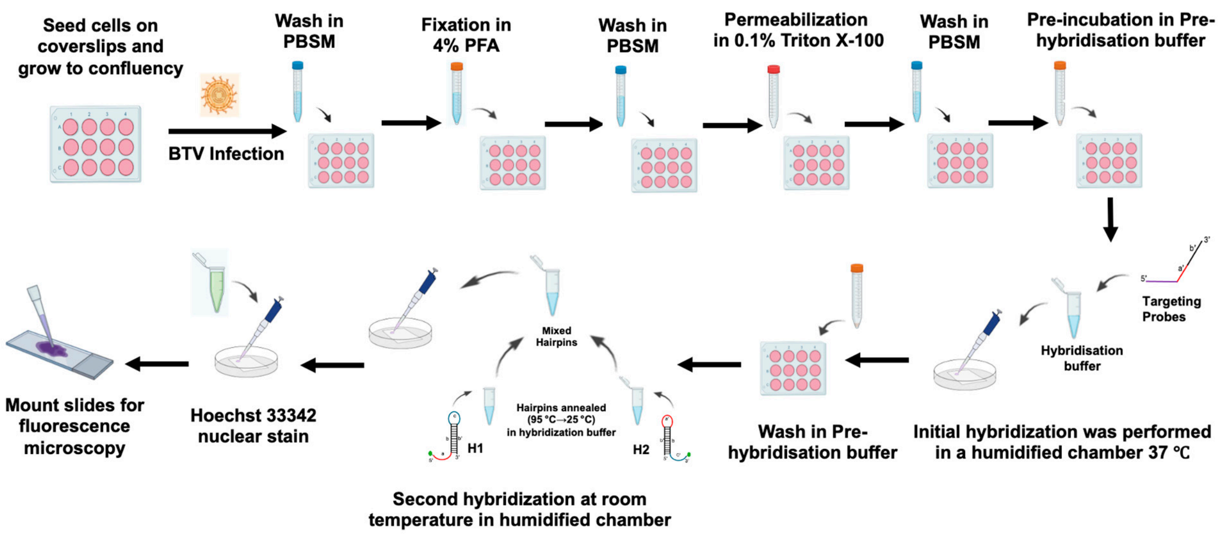

Figure S2. The illustration depicts a schematic overview of the *in-situ* hybridization chain reaction (HCR) experiments conducted.

Table S1. RNA probes used for *in-situ* Hybridization Chain Reaction

S1-HCR-probes-atto488	Probe (5'-> 3')	Probe position	Percent GC
RNA-HCR-Probe-H1_5'-Atto488	Atto488 - 5' - TAGACTGAACCCACTCCGACG***ATCTGTCTT***CGTCGGAGTGGG - 3'		
RNA-HCR-Probe-H2_3'-Atto488	5' - CGTCGGAGTGGG***TTCAGTCTA***CCCACTCCGACGAAGACAGAT - 3' - Atto488		
BTV1-WT-S1_RNA-HCR-target_1	aacctcatctaatactggtatc***AAA***CGTCGGAGTGGGTTCAGTCTA	227	36.00%
BTV1-WT-S1_RNA-HCR-target_2	cctcaaaagcgtaactttggaa***AAA***CGTCGGAGTGGGTTCAGTCTA	279	41.00%
BTV1-WT-S1_RNA-HCR-target_3	tctcccttgaaactctataatt***AAA***CGTCGGAGTGGGTTCAGTCTA	360	32.00%
BTV1-WT-S1_RNA-HCR-target_4	ttaatgggtatatctccgtata***AAA***CGTCGGAGTGGGTTCAGTCTA	433	32.00%
BTV1-WT-S1_RNA-HCR-target_5	gcactcagttcattgatgaaac***AAA***CGTCGGAGTGGGTTCAGTCTA	466	41.00%
BTV1-WT-S1_RNA-HCR-target_6	tggaatggtgaatcgaaacggt***AAA***CGTCGGAGTGGGTTCAGTCTA	538	45.00%
BTV1-WT-S1_RNA-HCR-target_7	ttacaaaccaagagtatggtgc***AAA***CGTCGGAGTGGGTTCAGTCTA	798	41.00%
BTV1-WT-S1_RNA-HCR-target_8	aaacacatcctttgaattcctg***AAA***CGTCGGAGTGGGTTCAGTCTA	878	36.00%
BTV1-WT-S1_RNA-HCR-target_9	aatagggctttgtatcgatttg***AAA***CGTCGGAGTGGGTTCAGTCTA	922	36.00%
BTV1-WT-S1_RNA-HCR-target_10	aatggtgtagtataaaccgtct***AAA***CGTCGGAGTGGGTTCAGTCTA	1066	36.00%
BTV1-WT-S1_RNA-HCR-target_11	cttggaccaaatctctttttta***AAA***CGTCGGAGTGGGTTCAGTCTA	1384	32.00%
BTV1-WT-S1_RNA-HCR-target_12	aagccttaatccttgaattgat***AAA***CGTCGGAGTGGGTTCAGTCTA	1431	32.00%
BTV1-WT-S1_RNA-HCR-target_13	tcagtgaaaacagtatgtcctt***AAA***CGTCGGAGTGGGTTCAGTCTA	1468	36.00%
BTV1-WT-S1_RNA-HCR-target_14	agtttggtatagttctacacta***AAA***CGTCGGAGTGGGTTCAGTCTA	1514	32.00%
BTV1-WT-S1_RNA-HCR-target_15	caattgtgaatatatcgcggtc***AAA***CGTCGGAGTGGGTTCAGTCTA	1767	41.00%
BTV1-WT-S1_RNA-HCR-target_16	gcataccggttcgaaaattatg***AAA***CGTCGGAGTGGGTTCAGTCTA	1827	41.00%
BTV1-WT-S1_RNA-HCR-target_17	tgtagttttaaacagtcttcgc***AAA***CGTCGGAGTGGGTTCAGTCTA	1973	36.00%
BTV1-WT-S1_RNA-HCR-target_18	caatactcgatactggaagcac***AAA***CGTCGGAGTGGGTTCAGTCTA	2061	45.00%
BTV1-WT-S1_RNA-HCR-target_19	attaaaaattgaagccgccact***AAA***CGTCGGAGTGGGTTCAGTCTA	2357	36.00%
BTV1-WT-S1_RNA-HCR-target_20	taagcattaatattagctgcgc***AAA***CGTCGGAGTGGGTTCAGTCTA	2607	36.00%
BTV1-WT-S1_RNA-HCR-target_21	gaatctgtatcaacgtaaaccc***AAA***CGTCGGAGTGGGTTCAGTCTA	2721	41.00%
BTV1-WT-S1_RNA-HCR-target_22	cataacgatattaagagctgcc***AAA***CGTCGGAGTGGGTTCAGTCTA	2798	41.00%
BTV1-WT-S1_RNA-HCR-target_23	tgatattctagttcgtaaccac***AAA***CGTCGGAGTGGGTTCAGTCTA	3136	36.00%
BTV1-WT-S1_RNA-HCR-target_24	agatgttgatattactcctgac***AAA***CGTCGGAGTGGGTTCAGTCTA	3209	36.00%
BTV1-WT-S1_RNA-HCR-target_25	ccttcgaagtatacgtcaaaga***AAA***CGTCGGAGTGGGTTCAGTCTA	3241	41.00%
BTV1-WT-S1_RNA-HCR-target_26	gataagttctggtctggaaaca***AAA***CGTCGGAGTGGGTTCAGTCTA	3286	41.00%
BTV1-WT-S1_RNA-HCR-target_27	caatatcacatcaatccgatct***AAA***CGTCGGAGTGGGTTCAGTCTA	3377	36.00%
BTV1-WT-S1_RNA-HCR-target_28	tctcaattacgttcagaatggt***AAA***CGTCGGAGTGGGTTCAGTCTA	3438	36.00%
BTV1-WT-S1_RNA-HCR-target_29	ttttcgtcaactgatagggata***AAA***CGTCGGAGTGGGTTCAGTCTA	3672	36.00%
BTV1-WT-S1_RNA-HCR-target_30	actttaattttaggtacgtgcg***AAA***CGTCGGAGTGGGTTCAGTCTA	3835	36.00%

S6-HCR-probes-cy3	Probe (5'-> 3')	Probe position	Percent GC
RNA-HCR-Probe-H1_5'-cy3	cy3 - 5' - AGTACATGTCGTGGTGGTAGC***TTGTATGAA***GCTACCACCACG - 3'		
RNA-HCR-Probe-H2_3'-cy3	5' - GCTACCACCACG***ACATGTACT***CGTGGTGGTAGCTTCATACAA - 3' - cy3		
BTV1-WT-S6_RNA-HCR-target_1	gtggttgccaactagagaac***AAA***GCTACCACCACGACATGTACT	10	50.00%
BTV1-WT-S6_RNA-HCR-target_2	gccaaaaaagttctcgtggc***AAA***GCTACCACCACGACATGTACT	83	50.00%
BTV1-WT-S6_RNA-HCR-target_3	atgactgcaagtccattgtg***AAA***GCTACCACCACGACATGTACT	111	45.00%
BTV1-WT-S6_RNA-HCR-target_4	aatttatatgctttcgccgg***AAA***GCTACCACCACGACATGTACT	215	40.00%
BTV1-WT-S6_RNA-HCR-target_5	ttttgaagcattggagccag***AAA***GCTACCACCACGACATGTACT	277	45.00%
BTV1-WT-S6_RNA-HCR-target_6	cggtaatcttcgagcagttg***AAA***GCTACCACCACGACATGTACT	353	50.00%
BTV1-WT-S6_RNA-HCR-target_7	taaccaatgcggattgcttc***AAA***GCTACCACCACGACATGTACT	400	45.00%
BTV1-WT-S6_RNA-HCR-target_8	gttggattcacgatttgacc***AAA***GCTACCACCACGACATGTACT	488	45.00%
BTV1-WT-S6_RNA-HCR-target_9	gcatccgggttgtagaaata***AAA***GCTACCACCACGACATGTACT	539	45.00%
BTV1-WT-S6_RNA-HCR-target_10	tctcaacctcacgtttgatt***AAA***GCTACCACCACGACATGTACT	613	40.00%
BTV1-WT-S6_RNA-HCR-target_11	cagtgtagggacatgtgtta***AAA***GCTACCACCACGACATGTACT	640	45.00%
BTV1-WT-S6_RNA-HCR-target_12	gatcagctgaatcggcaaga***AAA***GCTACCACCACGACATGTACT	690	50.00%
BTV1-WT-S6_RNA-HCR-target_13	aatattgctgtatcgccatc***AAA***GCTACCACCACGACATGTACT	760	40.00%
BTV1-WT-S6_RNA-HCR-target_14	atctgacctcttcagcataa***AAA***GCTACCACCACGACATGTACT	790	40.00%
BTV1-WT-S6_RNA-HCR-target_15	cgttggaacccttccaaaaa***AAA***GCTACCACCACGACATGTACT	822	45.00%
BTV1-WT-S6_RNA-HCR-target_16	gttgggaaatcgcgtcttat***AAA***GCTACCACCACGACATGTACT	872	45.00%
BTV1-WT-S6_RNA-HCR-target_17	taatcataggtagcagccag***AAA***GCTACCACCACGACATGTACT	952	45.00%
BTV1-WT-S6_RNA-HCR-target_18	gctgacatgtatgctttcta***AAA***GCTACCACCACGACATGTACT	1030	40.00%
BTV1-WT-S6_RNA-HCR-target_19	gatctgctttgagtgtttca***AAA***GCTACCACCACGACATGTACT	1059	40.00%
BTV1-WT-S6_RNA-HCR-target_20	tgatgcgcgtacatcaatca***AAA***GCTACCACCACGACATGTACT	1092	45.00%
BTV1-WT-S6_RNA-HCR-target_21	gaatcatttcccacatgttc***AAA***GCTACCACCACGACATGTACT	1154	40.00%
BTV1-WT-S6_RNA-HCR-target_22	cggtgaatgtgaatcgcagt***AAA***GCTACCACCACGACATGTACT	1274	50.00%
BTV1-WT-S6_RNA-HCR-target_23	cgcgtccgagcatgaaaata***AAA***GCTACCACCACGACATGTACT	1342	50.00%
BTV1-WT-S6_RNA-HCR-target_24	ccgtagcacacaaagcagaa***AAA***GCTACCACCACGACATGTACT	1415	50.00%
BTV1-WT-S6_RNA-HCR-target_25	tccggtcctttcaaaatgat***AAA***GCTACCACCACGACATGTACT	1496	40.00%
BTV1-WT-S6_RNA-HCR-target_26	aaacatcgtagcataagccc***AAA***GCTACCACCACGACATGTACT	1539	45.00%
BTV1-WT-S6_RNA-HCR-target_27	gggtgataatgcatcgaacc***AAA***GCTACCACCACGACATGTACT	1564	50.00%
BTV1-WT-S6_RNA-HCR-target_28	cagcgaagtgaacctttttct***AAA***GCTACCACCACGACATGTACT	1594	45.00%
BTV1-WT-S6_RNA-HCR-target_29	agcgagattgataacttccc***AAA***GCTACCACCACGACATGTACT	1641	45.00%
BTV1-WT-S6_RNA-HCR-target_30	aatactccatccacatctga***AAA***GCTACCACCACGACATGTACT	1672	40.00%

S10-HCR-probes-cy5	Probe (5'-> 3')	Probe position	Percent GC
RNA-HCR-Probe-H1_5'-cy5	cy5 - 5' - TGTTGCAAAGGAACGTCGAGC***TGTAATGGT***GCTCGACGTTCC - 3'		
RNA-HCR-Probe-H2_3'-cy5	5' - GCTCGACGTTCC***TTTGCAACA***GGAACGTCGAGCACCATTACA - 3' - cy5		
BTV1-WT-S10_RNA-HCR-target_1	atggcagcgacacttttt***AAA***GCTCGACGTTCCTTTGCAACA	4	44.00%
BTV1-WT-S10_RNA-HCR-target_2	tttggatcagcccggata***AAA***GCTCGACGTTCCTTTGCAACA	24	50.00%
BTV1-WT-S10_RNA-HCR-target_3	tcatttttcttcctcga***AAA***GCTCGACGTTCCTTTGCAACA	45	33.00%
BTV1-WT-S10_RNA-HCR-target_4	ttcaacccgttcctgatt***AAA***GCTCGACGTTCCTTTGCAACA	68	44.00%
BTV1-WT-S10_RNA-HCR-target_5	acacggaccagactcagc***AAA***GCTCGACGTTCCTTTGCAACA	88	61.00%
BTV1-WT-S10_RNA-HCR-target_6	gcggttgggaaatcgtgt***AAA***GCTCGACGTTCCTTTGCAACA	111	56.00%
BTV1-WT-S10_RNA-HCR-target_7	cgcactcggagcatatct***AAA***GCTCGACGTTCCTTTGCAACA	131	56.00%
BTV1-WT-S10_RNA-HCR-target_8	aggcatcgacgatggcat***AAA***GCTCGACGTTCCTTTGCAACA	152	56.00%
BTV1-WT-S10_RNA-HCR-target_9	gctttgtccaagatttca***AAA***GCTCGACGTTCCTTTGCAACA	181	39.00%
BTV1-WT-S10_RNA-HCR-target_10	cacccgttgtatttgaca***AAA***GCTCGACGTTCCTTTGCAACA	201	44.00%
BTV1-WT-S10_RNA-HCR-target_11	cgctttttgtgtttgcgt***AAA***GCTCGACGTTCCTTTGCAACA	221	44.00%
BTV1-WT-S10_RNA-HCR-target_12	gatgcgaatgcagccttc***AAA***GCTCGACGTTCCTTTGCAACA	241	56.00%
BTV1-WT-S10_RNA-HCR-target_13	cacgaaacgcttctgcgt***AAA***GCTCGACGTTCCTTTGCAACA	261	56.00%
BTV1-WT-S10_RNA-HCR-target_14	ctgtctcaacctcacgtc***AAA***GCTCGACGTTCCTTTGCAACA	281	56.00%
BTV1-WT-S10_RNA-HCR-target_15	ctttaagcctcctaggtc***AAA***GCTCGACGTTCCTTTGCAACA	344	50.00%
BTV1-WT-S10_RNA-HCR-target_16	aacaaccgccgctatcag***AAA***GCTCGACGTTCCTTTGCAACA	392	56.00%
BTV1-WT-S10_RNA-HCR-target_17	agcttaaacgccacgctc***AAA***GCTCGACGTTCCTTTGCAACA	448	56.00%
BTV1-WT-S10_RNA-HCR-target_18	ccattgaggtatctcagc***AAA***GCTCGACGTTCCTTTGCAACA	479	50.00%
BTV1-WT-S10_RNA-HCR-target_19	cattgggttcagactctt***AAA***GCTCGACGTTCCTTTGCAACA	500	44.00%
BTV1-WT-S10_RNA-HCR-target_20	cccaaattcaccacacct***AAA***GCTCGACGTTCCTTTGCAACA	520	50.00%
BTV1-WT-S10_RNA-HCR-target_21	gaccatcatcaggaaggt***AAA***GCTCGACGTTCCTTTGCAACA	542	50.00%
BTV1-WT-S10_RNA-HCR-target_22	cttctctcgctttttgcg***AAA***GCTCGACGTTCCTTTGCAACA	562	50.00%
BTV1-WT-S10_RNA-HCR-target_23	tgtcaatttgctggttca***AAA***GCTCGACGTTCCTTTGCAACA	582	39.00%
BTV1-WT-S10_RNA-HCR-target_24	tcttcatcacttccttct***AAA***GCTCGACGTTCCTTTGCAACA	606	39.00%
BTV1-WT-S10_RNA-HCR-target_25	tcctcaccgcatcattat***AAA***GCTCGACGTTCCTTTGCAACA	633	44.00%
BTV1-WT-S10_RNA-HCR-target_26	ccatctagcgggactgat***AAA***GCTCGACGTTCCTTTGCAACA	670	56.00%
BTV1-WT-S10_RNA-HCR-target_27	gccactctacctactgat***AAA***GCTCGACGTTCCTTTGCAACA	711	50.00%
BTV1-WT-S10_RNA-HCR-target_28	acacgatgcagacctcgg***AAA***GCTCGACGTTCCTTTGCAACA	732	61.00%
BTV1-WT-S10_RNA-HCR-target_29	gtctgcatcgtgagatca***AAA***GCTCGACGTTCCTTTGCAACA	758	50.00%
BTV1-WT-S10_RNA-HCR-target_30	cccgttagacagcagtag***AAA***GCTCGACGTTCCTTTGCAACA	778	56.00%

S6-HCR-probes-cy5	Probe (5'-> 3')	Probe position	Percent GC
RNA-HCR-Probe-H1_5'-cy5	cy5 - 5' - TGTTGCAAAGGAACGTCGAGC***TGTAATGGT***GCTCGACGTTCC - 3'		
RNA-HCR-Probe-H2_3'-cy5	5' - GCTCGACGTTCC***TTTGCAACA***GGAACGTCGAGCACCATTACA - 3' - cy5		
BTV1-WT-S6_RNA-HCR-target_1	gtggttgccaactagagaac***AAA***GCTCGACGTTCCTTTGCAACA	10	50.00%
BTV1-WT-S6_RNA-HCR-target_2	gccaaaaaagttctcgtggc***AAA***GCTCGACGTTCCTTTGCAACA	83	50.00%
BTV1-WT-S6_RNA-HCR-target_3	atgactgcaagtccattgtg***AAA***GCTCGACGTTCCTTTGCAACA	111	45.00%
BTV1-WT-S6_RNA-HCR-target_4	aatttatatgctttcgccgg***AAA***GCTCGACGTTCCTTTGCAACA	215	40.00%
BTV1-WT-S6_RNA-HCR-target_5	ttttgaagcattggagccag***AAA***GCTCGACGTTCCTTTGCAACA	277	45.00%
BTV1-WT-S6_RNA-HCR-target_6	cggtaatcttcgagcagttg***AAA***GCTCGACGTTCCTTTGCAACA	353	50.00%
BTV1-WT-S6_RNA-HCR-target_7	taaccaatgcggattgcttc***AAA***GCTCGACGTTCCTTTGCAACA	400	45.00%
BTV1-WT-S6_RNA-HCR-target_8	gttggattcacgatttgacc***AAA***GCTCGACGTTCCTTTGCAACA	488	45.00%
BTV1-WT-S6_RNA-HCR-target_9	gcatccgggttgtagaaata***AAA***GCTCGACGTTCCTTTGCAACA	539	45.00%
BTV1-WT-S6_RNA-HCR-target_10	tctcaacctcacgtttgatt***AAA***GCTCGACGTTCCTTTGCAACA	613	40.00%
BTV1-WT-S6_RNA-HCR-target_11	cagtgtagggacatgtgtta***AAA***GCTCGACGTTCCTTTGCAACA	640	45.00%
BTV1-WT-S6_RNA-HCR-target_12	gatcagctgaatcggcaaga***AAA***GCTCGACGTTCCTTTGCAACA	690	50.00%
BTV1-WT-S6_RNA-HCR-target_13	aatattgctgtatcgccatc***AAA***GCTCGACGTTCCTTTGCAACA	760	40.00%
BTV1-WT-S6_RNA-HCR-target_14	atctgacctcttcagcataa***AAA***GCTCGACGTTCCTTTGCAACA	790	40.00%
BTV1-WT-S6_RNA-HCR-target_15	cgttggaacccttccaaaaa***AAA***GCTCGACGTTCCTTTGCAACA	822	45.00%
BTV1-WT-S6_RNA-HCR-target_16	gttgggaaatcgcgtcttat***AAA***GCTCGACGTTCCTTTGCAACA	872	45.00%
BTV1-WT-S6_RNA-HCR-target_17	taatcataggtagcagccag***AAA***GCTCGACGTTCCTTTGCAACA	952	45.00%
BTV1-WT-S6_RNA-HCR-target_18	gctgacatgtatgctttcta***AAA***GCTCGACGTTCCTTTGCAACA	1030	40.00%
BTV1-WT-S6_RNA-HCR-target_19	gatctgctttgagtgtttca***AAA***GCTCGACGTTCCTTTGCAACA	1059	40.00%
BTV1-WT-S6_RNA-HCR-target_20	tgatgcgcgtacatcaatca***AAA***GCTCGACGTTCCTTTGCAACA	1092	45.00%
BTV1-WT-S6_RNA-HCR-target_21	gaatcatttcccacatgttc***AAA***GCTCGACGTTCCTTTGCAACA	1154	40.00%
BTV1-WT-S6_RNA-HCR-target_22	cggtgaatgtgaatcgcagt***AAA***GCTCGACGTTCCTTTGCAACA	1274	50.00%
BTV1-WT-S6_RNA-HCR-target_23	cgcgtccgagcatgaaaata***AAA***GCTCGACGTTCCTTTGCAACA	1342	50.00%
BTV1-WT-S6_RNA-HCR-target_24	ccgtagcacacaaagcagaa***AAA***GCTCGACGTTCCTTTGCAACA	1415	50.00%
BTV1-WT-S6_RNA-HCR-target_25	tccggtcctttcaaaatgat***AAA***GCTCGACGTTCCTTTGCAACA	1496	40.00%
BTV1-WT-S6_RNA-HCR-target_26	aaacatcgtagcataagccc***AAA***GCTCGACGTTCCTTTGCAACA	1539	45.00%
BTV1-WT-S6_RNA-HCR-target_27	gggtgataatgcatcgaacc***AAA***GCTCGACGTTCCTTTGCAACA	1564	50.00%
BTV1-WT-S6_RNA-HCR-target_28	cagcgaagtgaaccttttct***AAA***GCTCGACGTTCCTTTGCAACA	1594	45.00%
BTV1-WT-S6_RNA-HCR-target_29	agcgagattgataacttccc***AAA***GCTCGACGTTCCTTTGCAACA	1641	45.00%
BTV1-WT-S6_RNA-HCR-target_30	aatactccatccacatctga***AAA***GCTCGACGTTCCTTTGCAACA	1672	40.00%

S1-HCR-probes-cy5	Probe (5'-> 3')	Probe position	Percent GC
RNA-HCR-Probe-H1_5'-cy5	cy5 - 5' - TGTTGCAAAGGAACGTCGAGC***TGTAATGGT***GCTCGACGTTCC - 3'		
RNA-HCR-Probe-H2_3'-cy5	5' - GCTCGACGTTCC***TTTGCAACA***GGAACGTCGAGCACCATTACA - 3' - cy5		
BTV1-WT-S1_RNA-HCR-target_1	aacctcatctaatactggtatc***AAA***GCTCGACGTTCCTTTGCAACA	227	36.00%
BTV1-WT-S1_RNA-HCR-target_2	cctcaaaagcgtaactttggaa***AAA***GCTCGACGTTCCTTTGCAACA	279	41.00%
BTV1-WT-S1_RNA-HCR-target_3	tctcccttgaaactctataatt***AAA***GCTCGACGTTCCTTTGCAACA	360	32.00%
BTV1-WT-S1_RNA-HCR-target_4	ttaatgggtatatctccgtata***AAA***GCTCGACGTTCCTTTGCAACA	433	32.00%
BTV1-WT-S1_RNA-HCR-target_5	gcactcagttcattgatgaaac***AAA***GCTCGACGTTCCTTTGCAACA	466	41.00%
BTV1-WT-S1_RNA-HCR-target_6	tggaatggtgaatcgaaacggt***AAA***GCTCGACGTTCCTTTGCAACA	538	45.00%
BTV1-WT-S1_RNA-HCR-target_7	ttacaaaccaagagtatggtgc***AAA***GCTCGACGTTCCTTTGCAACA	798	41.00%
BTV1-WT-S1_RNA-HCR-target_8	aaacacatcctttgaattcctg***AAA***GCTCGACGTTCCTTTGCAACA	878	36.00%
BTV1-WT-S1_RNA-HCR-target_9	aatagggctttgtatcgatttg***AAA***GCTCGACGTTCCTTTGCAACA	922	36.00%
BTV1-WT-S1_RNA-HCR-target_10	aatggtgtagtataaaccgtct***AAA***GCTCGACGTTCCTTTGCAACA	1066	36.00%
BTV1-WT-S1_RNA-HCR-target_11	cttggaccaaatctctttttta***AAA***GCTCGACGTTCCTTTGCAACA	1384	32.00%
BTV1-WT-S1_RNA-HCR-target_12	aagccttaatccttgaattgat***AAA***GCTCGACGTTCCTTTGCAACA	1431	32.00%
BTV1-WT-S1_RNA-HCR-target_13	tcagtgaaaacagtatgtcctt***AAA***GCTCGACGTTCCTTTGCAACA	1468	36.00%
BTV1-WT-S1_RNA-HCR-target_14	agtttggtatagttctacacta***AAA***GCTCGACGTTCCTTTGCAACA	1514	32.00%
BTV1-WT-S1_RNA-HCR-target_15	caattgtgaatatatcgcggtc***AAA***GCTCGACGTTCCTTTGCAACA	1767	41.00%
BTV1-WT-S1_RNA-HCR-target_16	gcataccggttcgaaaattatg***AAA***GCTCGACGTTCCTTTGCAACA	1827	41.00%
BTV1-WT-S1_RNA-HCR-target_17	tgtagttttaaacagtcttcgc***AAA***GCTCGACGTTCCTTTGCAACA	1973	36.00%
BTV1-WT-S1_RNA-HCR-target_18	caatactcgatactggaagcac***AAA***GCTCGACGTTCCTTTGCAACA	2061	45.00%
BTV1-WT-S1_RNA-HCR-target_19	attaaaaattgaagccgccact***AAA***GCTCGACGTTCCTTTGCAACA	2357	36.00%
BTV1-WT-S1_RNA-HCR-target_20	taagcattaatattagctgcgc***AAA***GCTCGACGTTCCTTTGCAACA	2607	36.00%
BTV1-WT-S1_RNA-HCR-target_21	gaatctgtatcaacgtaaaccc***AAA***GCTCGACGTTCCTTTGCAACA	2721	41.00%
BTV1-WT-S1_RNA-HCR-target_22	cataacgatattaagagctgcc***AAA***GCTCGACGTTCCTTTGCAACA	2798	41.00%
BTV1-WT-S1_RNA-HCR-target_23	tgatattctagttcgtaaccac***AAA***GCTCGACGTTCCTTTGCAACA	3136	36.00%
BTV1-WT-S1_RNA-HCR-target_24	agatgttgatattactcctgac***AAA***GCTCGACGTTCCTTTGCAACA	3209	36.00%
BTV1-WT-S1_RNA-HCR-target_25	ccttcgaagtatacgtcaaaga***AAA***GCTCGACGTTCCTTTGCAACA	3241	41.00%
BTV1-WT-S1_RNA-HCR-target_26	gataagttctggtctggaaaca***AAA***GCTCGACGTTCCTTTGCAACA	3286	41.00%
BTV1-WT-S1_RNA-HCR-target_27	caatatcacatcaatccgatct***AAA***GCTCGACGTTCCTTTGCAACA	3377	36.00%
BTV1-WT-S1_RNA-HCR-target_28	tctcaattacgttcagaatggt***AAA***GCTCGACGTTCCTTTGCAACA	3438	36.00%
BTV1-WT-S1_RNA-HCR-target_29	ttttcgtcaactgatagggata***AAA***GCTCGACGTTCCTTTGCAACA	3672	36.00%
BTV1-WT-S1_RNA-HCR-target_30	actttaattttaggtacgtgcg***AAA***GCTCGACGTTCCTTTGCAACA	3835	36.00%

ActB-HCR-probes-Cy5	Probe (5'-> 3')	Probe position	Percent GC
RNA-HCR-Probe-H1_5'-cy5	cy5 - 5' - TGTTGCAAAGGAACGTCGAGC***TGTAATGGT***GCTCGACGTTCC - 3'		
RNA-HCR-Probe-H2_3'-cy5	5' - GCTCGACGTTCC***TTTGCAACA***GGAACGTCGAGCACCATTACA - 3' - cy5		
BHK-21_ actb-mRNA-HCR-target_1	acaacgagcgcagcgatatc**AAA**GCTCGACGTTCCTTTGCAACA	10	55.00%
BHK-21_ actb-mRNA-HCR-target_2	cacgatggaggggaagacgg**AAA**GCTCGACGTTCCTTTGCAACA	86	65.00%
BHK-21_ actb-mRNA-HCR-target_3	agaatacctctcttgctctg**AAA**GCTCGACGTTCCTTTGCAACA	175	45.00%
BHK-21_ actb-mRNA-HCR-target_4	gtgacaatgccgtgttcaat**AAA**GCTCGACGTTCCTTTGCAACA	211	45.00%
BHK-21_ actb-mRNA-HCR-target_5	cagatcttctccatatcgtc**AAA**GCTCGACGTTCCTTTGCAACA	238	45.00%
BHK-21_ actb-mRNA-HCR-target_6	cacgcagctcgttgtagaag**AAA**GCTCGACGTTCCTTTGCAACA	267	55.00%
BHK-21_ actb-mRNA-HCR-target_7	tgggtcatcttttcacggtt**AAA**GCTCGACGTTCCTTTGCAACA	343	45.00%
BHK-21_ actb-mRNA-HCR-target_8	ggtgttgaaggtctcaaaca**AAA**GCTCGACGTTCCTTTGCAACA	368	45.00%
BHK-21_ actb-mRNA-HCR-target_9	cctgaatggctacgtacatg**AAA**GCTCGACGTTCCTTTGCAACA	393	50.00%
BHK-21_ actb-mRNA-HCR-target_10	gtacgaccagaggcatacag**AAA**GCTCGACGTTCCTTTGCAACA	424	55.00%
BHK-21_ actb-mRNA-HCR-target_11	ctccggagtccatcacaatg**AAA**GCTCGACGTTCCTTTGCAACA	450	55.00%
BHK-21_ actb-mRNA-HCR-target_12	tcatagatgggcacagtgtg**AAA**GCTCGACGTTCCTTTGCAACA	481	50.00%
BHK-21_ actb-mRNA-HCR-target_13	caggatggcatgagggagag**AAA**GCTCGACGTTCCTTTGCAACA	509	60.00%
BHK-21_ actb-mRNA-HCR-target_14	atcttcatgaggtagtctgt**AAA**GCTCGACGTTCCTTTGCAACA	556	40.00%
BHK-21_ actb-mRNA-HCR-target_15	ctgtggtggtgaagctgtag**AAA**GCTCGACGTTCCTTTGCAACA	591	55.00%
BHK-21_ actb-mRNA-HCR-target_16	ctctttgatgtcacgcacaa**AAA**GCTCGACGTTCCTTTGCAACA	623	45.00%
BHK-21_ actb-mRNA-HCR-target_17	cgaagtccagggcaacatag**AAA**GCTCGACGTTCCTTTGCAACA	651	55.00%
BHK-21_ actb-mRNA-HCR-target_18	ctccagggaggaagaggatg**AAA**GCTCGACGTTCCTTTGCAACA	692	60.00%
BHK-21_ actb-mRNA-HCR-target_19	tcgttgccaatggtgatgac**AAA**GCTCGACGTTCCTTTGCAACA	739	50.00%
BHK-21_ actb-mRNA-HCR-target_20	gaaaagggcctcagggcaac**AAA**GCTCGACGTTCCTTTGCAACA	767	60.00%
BHK-21_ actb-mRNA-HCR-target_21	attccatacccaggaaggaa**AAA**GCTCGACGTTCCTTTGCAACA	792	45.00%
BHK-21_ actb-mRNA-HCR-target_22	aatgtagtttcgtggatgcc**AAA**GCTCGACGTTCCTTTGCAACA	817	45.00%
BHK-21_ actb-mRNA-HCR-target_23	gacgtcacacttcatgatgg**AAA**GCTCGACGTTCCTTTGCAACA	842	50.00%
BHK-21_ actb-mRNA-HCR-target_24	tgttggcatagaggtctttg**AAA**GCTCGACGTTCCTTTGCAACA	870	45.00%
BHK-21_ actb-mRNA-HCR-target_25	caatgcctgggtacatggtg**AAA**GCTCGACGTTCCTTTGCAACA	909	55.00%
BHK-21_ actb-mRNA-HCR-target_26	agagcagtgatctccttctg**AAA**GCTCGACGTTCCTTTGCAACA	940	50.00%
BHK-21_ actb-mRNA-HCR-target_27	ggggagcaatgatcttgatc**AAA**GCTCGACGTTCCTTTGCAACA	978	50.00%
BHK-21_ actb-mRNA-HCR-target_28	cgatccacacagagtacttg**AAA**GCTCGACGTTCCTTTGCAACA	1005	50.00%
BHK-21_ actb-mRNA-HCR-target_29	tgatccacatctgctggaag**AAA**GCTCGACGTTCCTTTGCAACA	1053	50.00%
BHK-21_ actb-mRNA-HCR-target_30	tagaagcatttgcggtggac**AAA**GCTCGACGTTCCTTTGCAACA	1108	50.00%

ActB-HCR-probes-Cy3	Probe (5'-> 3')	Probe position	Percent GC
RNA-HCR-Probe-H1_5'-cy3	cy3 - 5' - AGTACATGTCGTGGTGGTAGC***TTGTATGAA***GCTACCACCACG - 3'		
RNA-HCR-Probe-H2_3'-cy3	5' - GCTACCACCACG***ACATGTACT***CGTGGTGGTAGCTTCATACAA - 3' - cy3		
BHK-21_ actb-mRNA-HCR-target_1	acaacgagcgcagcgatatc**AAA**GCTACCACCACGACATGTACT	10	55.00%
BHK-21_ actb-mRNA-HCR-target_2	cacgatggaggggaagacgg**AAA**GCTACCACCACGACATGTACT	86	65.00%
BHK-21_ actb-mRNA-HCR-target_3	agaatacctctcttgctctg**AAA**GCTACCACCACGACATGTACT	175	45.00%
BHK-21_ actb-mRNA-HCR-target_4	gtgacaatgccgtgttcaat**AAA**GCTACCACCACGACATGTACT	211	45.00%
BHK-21_ actb-mRNA-HCR-target_5	cagatcttctccatatcgtc**AAA**GCTACCACCACGACATGTACT	238	45.00%
BHK-21_ actb-mRNA-HCR-target_6	cacgcagctcgttgtagaag**AAA**GCTACCACCACGACATGTACT	267	55.00%
BHK-21_ actb-mRNA-HCR-target_7	tgggtcatcttttcacggtt**AAA**GCTACCACCACGACATGTACT	343	45.00%
BHK-21_ actb-mRNA-HCR-target_8	ggtgttgaaggtctcaaaca**AAA**GCTACCACCACGACATGTACT	368	45.00%
BHK-21_ actb-mRNA-HCR-target_9	cctgaatggctacgtacatg**AAA**GCTACCACCACGACATGTACT	393	50.00%
BHK-21_ actb-mRNA-HCR-target_10	gtacgaccagaggcatacag**AAA**GCTACCACCACGACATGTACT	424	55.00%
BHK-21_ actb-mRNA-HCR-target_11	ctccggagtccatcacaatg**AAA**GCTACCACCACGACATGTACT	450	55.00%
BHK-21_ actb-mRNA-HCR-target_12	tcatagatgggcacagtgtg**AAA**GCTACCACCACGACATGTACT	481	50.00%
BHK-21_ actb-mRNA-HCR-target_13	caggatggcatgagggagag**AAA**GCTACCACCACGACATGTACT	509	60.00%
BHK-21_ actb-mRNA-HCR-target_14	atcttcatgaggtagtctgt**AAA**GCTACCACCACGACATGTACT	556	40.00%
BHK-21_ actb-mRNA-HCR-target_15	ctgtggtggtgaagctgtag**AAA**GCTACCACCACGACATGTACT	591	55.00%
BHK-21_ actb-mRNA-HCR-target_16	ctctttgatgtcacgcacaa**AAA**GCTACCACCACGACATGTACT	623	45.00%
BHK-21_ actb-mRNA-HCR-target_17	cgaagtccagggcaacatag**AAA**GCTACCACCACGACATGTACT	651	55.00%
BHK-21_ actb-mRNA-HCR-target_18	ctccagggaggaagaggatg**AAA**GCTACCACCACGACATGTACT	692	60.00%
BHK-21_ actb-mRNA-HCR-target_19	tcgttgccaatggtgatgac**AAA**GCTACCACCACGACATGTACT	739	50.00%
BHK-21_ actb-mRNA-HCR-target_20	gaaaagggcctcagggcaac**AAA**GCTACCACCACGACATGTACT	767	60.00%
BHK-21_ actb-mRNA-HCR-target_21	attccatacccaggaaggaa**AAA**GCTACCACCACGACATGTACT	792	45.00%
BHK-21_ actb-mRNA-HCR-target_22	aatgtagtttcgtggatgcc**AAA**GCTACCACCACGACATGTACT	817	45.00%
BHK-21_ actb-mRNA-HCR-target_23	gacgtcacacttcatgatgg**AAA**GCTACCACCACGACATGTACT	842	50.00%
BHK-21_ actb-mRNA-HCR-target_24	tgttggcatagaggtctttg**AAA**GCTACCACCACGACATGTACT	870	45.00%
BHK-21_ actb-mRNA-HCR-target_25	caatgcctgggtacatggtg**AAA**GCTACCACCACGACATGTACT	909	55.00%
BHK-21_ actb-mRNA-HCR-target_26	agagcagtgatctccttctg**AAA**GCTACCACCACGACATGTACT	940	50.00%
BHK-21_ actb-mRNA-HCR-target_27	ggggagcaatgatcttgatc**AAA**GCTACCACCACGACATGTACT	978	50.00%
BHK-21_ actb-mRNA-HCR-target_28	cgatccacacagagtacttg**AAA**GCTACCACCACGACATGTACT	1005	50.00%
BHK-21_ actb-mRNA-HCR-target_29	tgatccacatctgctggaag**AAA**GCTACCACCACGACATGTACT	1053	50.00%
BHK-21_ actb-mRNA-HCR-target_30	tagaagcatttgcggtggac**AAA**GCTACCACCACGACATGTACT	1108	50.00%

Positive-Control (S10_Cy3+Cy5)	Probe (5'-> 3')	Probe position	Percent GC
RNA-HCR-Probe-H1_5'-cy5	cy5 - 5' - TGTTGCAAAGGAACGTCGAGC***TGTAATGGT***GCTCGACGTTCC - 3'		
RNA-HCR-Probe-H2_3'-cy5	5' - GCTCGACGTTCC***TTTGCAACA***GGAACGTCGAGCACCATTACA - 3' - cy5		
BTV1-WT-S10_RNA-HCR-target_1	atggcagcgacactttt***AAA***GCTCGACGTTCCTTTGCAACA	4	44.00%
BTV1-WT-S10_RNA-HCR-target_2	tttggatcagcccggata***AAA***GCTCGACGTTCCTTTGCAACA	24	50.00%
BTV1-WT-S10_RNA-HCR-target_3	tcattttttcttcctcga***AAA***GCTCGACGTTCCTTTGCAACA	45	33.00%
BTV1-WT-S10_RNA-HCR-target_4	ttcaacccgttcctgatt***AAA***GCTCGACGTTCCTTTGCAACA	68	44.00%
BTV1-WT-S10_RNA-HCR-target_5	acacggaccagactcagc***AAA***GCTCGACGTTCCTTTGCAACA	88	61.00%
BTV1-WT-S10_RNA-HCR-target_6	gcggttgggaaatcgtgt***AAA***GCTCGACGTTCCTTTGCAACA	111	56.00%
BTV1-WT-S10_RNA-HCR-target_7	cgcactcggagcatatct***AAA***GCTCGACGTTCCTTTGCAACA	131	56.00%
BTV1-WT-S10_RNA-HCR-target_8	aggcatcgacgatggcat***AAA***GCTCGACGTTCCTTTGCAACA	152	56.00%
BTV1-WT-S10_RNA-HCR-target_9	gctttgtccaagatttca***AAA***GCTCGACGTTCCTTTGCAACA	181	39.00%
BTV1-WT-S10_RNA-HCR-target_10	cacccgttgtatttgaca***AAA***GCTCGACGTTCCTTTGCAACA	201	44.00%
BTV1-WT-S10_RNA-HCR-target_11	cgctttttgtgtttgcgt***AAA***GCTCGACGTTCCTTTGCAACA	221	44.00%
BTV1-WT-S10_RNA-HCR-target_12	gatgcgaatgcagccttc***AAA***GCTCGACGTTCCTTTGCAACA	241	56.00%
BTV1-WT-S10_RNA-HCR-target_13	cacgaaacgcttctgcgt***AAA***GCTCGACGTTCCTTTGCAACA	261	56.00%
BTV1-WT-S10_RNA-HCR-target_14	ctgtctcaacctcacgtc***AAA***GCTCGACGTTCCTTTGCAACA	281	56.00%
BTV1-WT-S10_RNA-HCR-target_15	ctttaagcctcctaggtc***AAA***GCTCGACGTTCCTTTGCAACA	344	50.00%
Positive-Control (S10_Cy3+Cy5)	Probe (5'-> 3')	Probe position	Percent GC
RNA-HCR-Probe-H1_5'-cy3	cy3 - 5' - AGTACATGTCGTGGTGGTAGC***TTGTATGAA***GCTACCACCACG - 3'		
RNA-HCR-Probe-H2_3'-cy3	5' - GCTACCACCACG***ACATGTACT***CGTGGTGGTAGCTTCATACAA - 3' - cy3		
BTV1-WT-S10_RNA-HCR-target_16	aacaaccgccgctatcag***AAA***GCTACCACCACGACATGTACT	392	56.00%
BTV1-WT-S10_RNA-HCR-target_17	agcttaaacgccacgctc***AAA***GCTACCACCACGACATGTACT	448	56.00%
BTV1-WT-S10_RNA-HCR-target_18	ccattgaggtatctcagc***AAA***GCTACCACCACGACATGTACT	479	50.00%
BTV1-WT-S10_RNA-HCR-target_19	cattgggttcagactctt***AAA***GCTACCACCACGACATGTACT	500	44.00%
BTV1-WT-S10_RNA-HCR-target_20	cccaaattcaccacacct***AAA***GCTACCACCACGACATGTACT	520	50.00%
BTV1-WT-S10_RNA-HCR-target_21	gaccatcatcaggaaggt***AAA***GCTACCACCACGACATGTACT	542	50.00%
BTV1-WT-S10_RNA-HCR-target_22	cttctctcgctttttgcg***AAA***GCTACCACCACGACATGTACT	562	50.00%
BTV1-WT-S10_RNA-HCR-target_23	tgtcaatttgctggttca***AAA***GCTACCACCACGACATGTACT	582	39.00%
BTV1-WT-S10_RNA-HCR-target_24	tcttcatcacttccttct***AAA***GCTACCACCACGACATGTACT	606	39.00%
BTV1-WT-S10_RNA-HCR-target_25	tcctcaccgcatcattat***AAA***GCTACCACCACGACATGTACT	633	44.00%
BTV1-WT-S10_RNA-HCR-target_26	ccatctagcgggactgat***AAA***GCTACCACCACGACATGTACT	670	56.00%
BTV1-WT-S10_RNA-HCR-target_27	gccactctacctactgat***AAA***GCTACCACCACGACATGTACT	711	50.00%
BTV1-WT-S10_RNA-HCR-target_28	acacgatgcagacctcgg***AAA***GCTACCACCACGACATGTACT	732	61.00%
BTV1-WT-S10_RNA-HCR-target_29	gtctgcatcgtgagatca***AAA***GCTACCACCACGACATGTACT	758	50.00%
BTV1-WT-S10_RNA-HCR-target_30	cccgttagacagcagtag***AAA***GCTACCACCACGACATGTACT	778	56.00%

Table S2. Biotinylated oligos for RNA complex pull-down assay

Name of oligo	Sequences	Locations
S1 bio 300R	5’ BiosG/AAAAAAAACCTCAAAAGCGTAACTTTGG	S1 nt300
S1 bio 2700R	5’ BiosG/AAAAAAACTTTCTGTCGCGATACATC	S1 nt2700
S1 bio 3700R	5’ BiosG/AAAAAAACTTCCGTTTTCGTCAACTG	S1 nt3700
S6 bio 300R	5’ BiosG/AAAAAAAAGCTTTTGAAGCATTGGAG	S6 nt300
S6 bio 850R	5’ BiosG/AAAAAAAAATTCGCCCGTTGGAACCC	S6 nt850
S10 bio 300R	5’ BiosG/AAAAAAAAATCTGTCTCAACCTCACGTC	S10 nt300
S10 bio 700R	5’ BiosG/AAAAAAAATGGTAATTCGAAACCATCTAG	S10 nt700

Table S3. qPCR primers for BTV ten segments

qPCR primers	Sequences	Size (nt)	GC (%)	TM (°C)	Target size (bp)
BTV1-wt_S1-F	5' TCGACACGCACCTTTCCGG 3'	19	63.16	62.9	109
BTV1-wt_S1-R	5' ATCCCTGGCTGCTCCTTCC 3'	19	63.16	61.7	
BTV1-wt_S2-F	5' ACATTTCATGCGGCGCAGGA 3'	20	55	61.3	109
BTV1-wt_S2-R	5' TCTGATCCCCTGGCCTAAACG 3'	21	57	64.1	
BTV1-wt_S3-F	5' GCCGACCCAGTCGTGCTAG 3'	19	68.42	62.5	109
BTV1-wt_S3-R	5' CGATCAGCGGAGCAATCTCG 3'	20	60	60.2	
BTV1-wt_S4-F	5' CCGTCTCGTTAAAGGAGCCG 3'	20	60	60.6	109
BTV1-wt_S4-R	5' GTTGGGTTTCGGACCCTCA 3'	19	57.89	60.7	
BTV1-wt_S5-F	5' ATGCGGAGACGGTCATGGTG 3'	20	60	61.8	109
BTV1-wt_S5-R	5' GCGATCTCCTGTATCGCCTCC 3'	21	61.9	64.3	
BTV1-wt_S6-F	5' GGATATGCGCCGCGTGCAG 3'	19	68.42	63	109
BTV1-wt_S6-R	5' CCCTCTCGTCCTCATCCTCG 3'	20	65	62	
BTV1-wt_S7-F	5' ACCAGCGCGTCAGCCCTAT 3'	19	63.16	62.4	109
BTV1-wt_S7-R	5' CGGACCACACACTACCGCA 3'	19	63.16	61.9	
BTV1-wt_S8-F	5' CGCCAAGGGAAGAGTCACGC 3'	20	65	63	109
BTV1-wt_S8-R	5' CTCGCTTCACGCAGCTTCTC 3'	20	60	60.2	
BTV1-wt_S9-F	5' CTGCTGAGAGAGGGAGGCG 3'	19	68.42	62.2	109
BTV1-wt_S9-R	5' GTGATCCCGACACTCGCTGG 3'	20	65	62.8	
BTV1-wt_S10-F	5' GCGCCTATGCCATCGTCG 3'	18	66.67	60.6	110
BTV1-wt_S10-R	5' GCGAATGCAGCCTTCTCCG 3'	19	63.16	61	
